# Human Immunodeficiency Viruses Pseudotyped with SARS-CoV-2 Spike Proteins Infect a Broad Spectrum of Human Cell Lines through Multiple Entry Mechanisms

**DOI:** 10.3390/v13060953

**Published:** 2021-05-21

**Authors:** Chuan Xu, Annie Wang, Ke Geng, William Honnen, Xuening Wang, Natalie Bruiners, Sukhwinder Singh, Fortunato Ferrara, Sara D’Angelo, Andrew R. M. Bradbury, Maria Laura Gennaro, Dongfang Liu, Abraham Pinter, Theresa L. Chang

**Affiliations:** 1 Public Health Research Institute, Rutgers, the State University of New Jersey, Newark, NJ 07103, USA; cx89@njms.rutgers.edu (C.X.); aw768@njms.rutgers.edu (A.W.); gengke@njms.rutgers.edu (K.G.); honnenwj@njms.rutgers.edu (W.H.); bruinena@njms.rutgers.edu (N.B.); gennarma@njms.rutgers.edu (M.L.G.); pinterab@njms.rutgers.edu (A.P.); 2 Laboratory Medicine, Rutgers, Department of Pathology, Immunology, the State University of New Jersey, Newark, NJ 07103, USA; xwang@njms.rutgers.edu (X.W.); dongfang.liu@rutgers.edu (D.L.); 3 NJMS Flow Cytometry and Immunology Core Laboratory, New Jersey Medical School, Rutgers, the State University of New Jersey, Newark, NJ 07103, USA; singhs1@njms.rutgers.edu; 4 Specifica Inc., Santa Fe, NM 87505, USA; fferrara@specifica.bio (F.F.); sdangelo@specifica.bio (S.D.); abradbury@specifica.bio (A.R.M.B.); 5 Department of Microbiology, Biochemistry and Molecular Genetics, Rutgers, the State University of New Jersey, Newark, NJ 07103, USA

**Keywords:** SARS-CoV-2 entry, hACE2, CD147, pseudotyped SARS-CoV-2 virus

## Abstract

Severe acute respiratory syndrome-related coronavirus (SARS-CoV-2), the causative agent of coronavirus disease 19 (COVID-19), enters cells through attachment to the human angiotensin converting enzyme 2 (hACE2) via the receptor-binding domain (RBD) in the surface/spike (S) protein. Several pseudotyped viruses expressing SARS-CoV-2 S proteins are available, but many of these can only infect hACE2-overexpressing cell lines. Here, we report the use of a simple, two-plasmid, pseudotyped virus system comprising a SARS-CoV-2 spike-expressing plasmid and an HIV vector with or without *vpr* to investigate the SARS-CoV-2 entry event in various cell lines. When an HIV vector without *vpr* was used, pseudotyped SARS-CoV-2 viruses produced in the presence of fetal bovine serum (FBS) were able to infect only engineered hACE2-overexpressing cell lines, whereas viruses produced under serum-free conditions were able to infect a broader range of cells, including cells without hACE2 overexpression. When an HIV vector containing *vpr* was used, pseudotyped viruses were able to infect a broad spectrum of cell types regardless of whether viruses were produced in the presence or absence of FBS. Infection sensitivities of various cell types did not correlate with mRNA abundance of hACE2, TMPRSS2, or TMPRSS4. Pseudotyped SARS-CoV-2 viruses and replication-competent SARS-CoV-2 virus were equally sensitive to neutralization by an anti-spike RBD antibody in cells with high abundance of hACE2. However, the anti-spike RBD antibody did not block pseudotyped viral entry into cell lines with low abundance of hACE2. We further found that CD147 was involved in viral entry in A549 cells with low abundance of hACE2. Thus, our assay is useful for drug and antibody screening as well as for investigating cellular receptors, including hACE2, CD147, and tyrosine-protein kinase receptor UFO (AXL), for the SARS-CoV-2 entry event in various cell lines.

## 1. Introduction

Severe acute respiratory syndrome-related coronavirus (SARS-CoV-2), the causative agent of coronavirus disease 19 (COVID-19), gains cellular entry primarily through binding of human angiotensin-converting enzyme 2 (hACE2) by surface (spike, S) proteins [1]. Other host factors, including TMPRSS2, TMPRSS4, neutrophilin-1, and heparan sulfate, interact with spike proteins and promote SARS-CoV-2 infection [1,2,3,4,5,6]. Although effective SARS-CoV-2 vaccines are available [7,8], potent anti-viral agents are still not clinically available to suppress infection in SARS-CoV-2-infected patients. A simple screening assay for assessing viral infectivity of different cell types would increase our understanding of viral permissiveness and would facilitate the process of drug discovery to combat this highly mutable virus.

Pseudotyped SARS-CoV-2 viruses expressing S proteins have been developed using various viral vectors, including HIV-1, murine leukemia virus, and vesicular stomatitis virus (VSV), and infection conditions have been optimized using cell lines that overexpress hACE2 or TMPRSS2, a serine protease that primes SARS-CoV-2 spike proteins for viral entry [1,9,10,11,12,13,14]. These cell lines have been useful for neutralizing antibody (Ab) studies. However, a system that relies on overexpression of hACE2 limits the potential for discovery of new receptors for viral entry. Here, we describe a simple, two-plasmid, HIV-1-based, pseudotyped virus that expresses full-length spike proteins and can infect cells that do not overexpress hACE2 receptors. This pseudotyped virus has the capacity to enter a broader range of human cell lines than standard pseudotyped viruses through hACE2 receptor-dependent and -independent means.

## 2. Materials and Methods

### 2.1. Reagents

The infectious-clone-derived SARS-CoV-2 virus (USA_WA1/2020 strain) expressing mNeonGreen was kindly provided by Pei-Yong Shi at the University of Texas Medical Branch, Galveston, TX, USA [15]. A recombinant construct used for infectivity assays (pseudotyped SARS-CoV-2) was derived from the full-length SARS-CoV-2-Wuhan-Hu-1 surface (spike) (GenBank accession number QHD43416) [16] which was codon optimized for humans and synthesized with Kozak-START GCCACC ATG and STOP codons and flanked by 5′ Nhel/3′Apal sites for subcloning into the pcDNA3.1(+) vector (Thermo Fisher Scientific, Waltham, MA, USA). HEK293T cells, Vero E6, A549, Calu-3, Caco-2, THP-1, HeLa, NCI-H1299, and BHK cells were purchased from American Type Culture Collection (ATCC). Intestinal epithelial cell lines, including WiDr, T84, SW480, and HT29, were kindly provided by Maria T. Abreu (University of Miami). The oral epithelial TR146 cell line and BL41 B-cell line were obtained from Daniel Fine (Rutgers, the State University of New Jersey) and George Miller (Yale University), respectively. SupT1 and MT4 CD4+ T-cell lines were obtained from the AIDS Research & Reference Reagent Program, National Institute of Allergy and Infectious Diseases, National Institutes of Health. hACE2-overexpressing HEK293T cells were kindly provided by Hyeryun Choe (Scripps Research, Jupiter, FL, USA) [17]. HeLa-hACE cells were provided by Dennis Burton (Scripps Research, La Jolla, CA, USA) [18]. Anti-CD147 Ab (clone# UM-8D6) with or without FITC conjugation and mouse IgG control Ab were purchased from Ancell (Bayport, MN, USA).

### 2.2. Cell Culture

Most adherent cells were cultured in Dulbecco’s Modified Eagle’s Medium (DMEM) supplemented with 10% fetal bovine serum (FBS). Vero E6 cells were cultured in Eagle’s Essential Minimal Medium (EMEM) with 5% FBS. TR146 cells were cultured in Ham’s F12 media with glutamate and 10% FBS. Suspension cells, including THP-1, SupT1, and MT4 cells, were maintained in RPMI-1640 with 10% FBS. PBMCs were isolated from the blood of healthy human donors obtained from the New York Blood Center by Histopaque^®^-1077 gradient centrifugation. Cells were activated with PHA (5 μg/mL) and IL-2 (50 IU/mL) for 3 days in RPMI-1640 with 10% FBS before infection.

### 2.3. Replication-Competent Virus Infection

Replication-competent SARS-CoV-2 viruses expressing mNeonGreen were propagated in Vero E6 cells as described previously [15]. All experiments were performed in a biosafety level 3 laboratory with personal protection equipment, including powered air purifying respirators (Breathe Easy, 3M), Tyvek suits, aprons, sleeves, booties, and double gloves. Virus titers were determined by plaque assays in Vero E6 cells as described previously [19]. For the infection assay, Vero E6 cells at 1 × 10^4^ cells per well were incubated overnight in a black 96-well glass plate (Greiner). Cells were exposed to viruses in the presence of serial dilutions of antibodies in 50 μL at a multiplicity of infection (MOI) of 5 for 1 h followed by the addition of 100 μL FluoroBrite medium containing 2% FBS. Fluorescence from productive viral infections and cell images was monitored at 24–48 h after infection using a Biotek Cytation 5.

### 2.4. Single-Cycle Infection with Pseudotyped Viruses

For single-cycle infection assays, replication-defective HIV-1 luciferase-expressing reporter viruses pseudotyped with SARS-CoV-2 S proteins (pseudotyped SARS-CoV-2 virus) were produced by co-transfection of a plasmid encoding the envelope-deficient HIV-1 NL4-3 virus with the luciferase reporter gene (pNL-4-3 Luc R-E- or pNL4-3 Luc R+E-, kindly provided by Nathaniel Landau, New York University) and a pcDNA3.1 plasmid expressing the full-length SARS-CoV-2 S protein into HEK 293T cells using Lipofectamine 3000. Specifically, 6–7 × 10^6^ cells were seeded and cultured overnight in a 100 mm^2^ dish. Lipofectamine 3000 (22 μL) in 500 μL of Opti-media was added to the mixture of the spike plasmid (7 μg), the HIV vector (7 μg), and P3000 (28 μL) in 500 μL of Opti-media according to the manufacture’s suggestion. The mixture was incubated for 15 min at room temperature before adding to the cells. The supernatant was collected 48 h after transfection and filtered. To produce SARS-CoV-2-pseudotyped viruses in the absence of serum, transfected cells were incubated for 24 h in medium with 10% FBS, and then the culture medium was replaced with DMEM without serum. Cells were cultured for an additional 24 h prior to collecting viruses. Viruses in the presence of 10% FBS harvested at 24 h were infectious, although the luciferase readout was ten-fold less than viruses harvested at 48 h. Virus stocks were analyzed for HIV-1 p24 antigen using the AlphaLISA HIV p24 kit (PerkinElmer, Waltham, MA, USA). Virus stocks contained approximately 200 ng/mL of HIV p24 protein. For pseudotyped viruses expressing GFP, HIV-1 vector pLai3ΔenvGFP3 [20] and SARS-CoV-2 S plasmid were used for transfection. The fluorescence of infected cells was monitored by flow cytometry (BD LSRII), by Echo Revolve microscopy, or by Biotek Cytation 5-cell imaging reader.

For infection assays, adherent cells were seeded at 5 × 10^4^ cells/well in a 48-well plate and cultured overnight. Suspension cells at 1.5 × 10^5^ per sample were used for infection. Pseudotyped SARS-CoV2 luciferase reporter viruses (100 μL, 20 ng of HIV p24 proteins) were added to cells for 1–2 h at 37 °C for attachment. Infected cells were cultured for 48–72 h, and HIV infection was determined by measuring luciferase activity (relative light units; RLUs) in lysed cells using Luciferase Substrate Buffer (Promega Inc., Madison, WI, USA) on a 2300 EnSpire Multilabel Plate Reader (PerkinElmer, Waltham, MA, USA).

### 2.5. Real-Time Quantitative RT-PCR

Total RNAs were extracted from cells using TRIzol (Life Technologies, Carlsbad, CA, USA). To synthesize first-strand cDNA, 2500 ng of total RNA, oligo(dT)12–18 (25 μg/mL), and dNTP (0.5 mM) were incubated at 65 °C for 5 min followed by quick chilling on ice. Reverse transcription was performed at 42 °C for 50 min and 70 °C for 15 min using SuperScript III Reverse Transcriptase. The PCR reaction contained cDNA equivalent to 125 ng of RNA input, 200 nM of primer sets, and SYBR Green Master Mix (QIAGEN), and was run in a StepOnePlus real-time PCR system (Life Technologies). PCR conditions included 95 °C denaturation for 10 min, 40 cycles of 95 °C for 15 s, and 60 °C for 60 s. PCR products were quantified and normalized relative to the amount of GAPDH cDNA products. Relative quantification of gene expression was calculated using the ΔΔCt (Ct, threshold cycle of real-time PCR) method according to the following formulas: ΔCT = Ct GAPDH − Ct target, ΔΔCt = ΔCt control − ΔCt target, using 2^−ΔΔCt^ method. The signals of various cell lines were then compared to HEK293T cells. Primer sequences were as follows: human GAPDH forward: (5′- GCA CCA CCA ACT GCT TAG CAC-3′); human GAPDH reverse: (5′- TCT TCT GGG TGG CAG TGA TG-3′); human ACE2 forward: (5′-CGA AGC CGA AGA CCT GTT CTA-3′); human ACE2 reverse: (5′-GGG CAA GTG TGG ACT GTT CC-3′); human TMPRSS2 forward: (5′- CAA GTG CTC CAA CTC TGG GAT-3′); human TMPRSS2 reverse: (5′- AAC ACA CCG ATT CTC GTC CTC-3′); human TMPRSS4 forward: (5′- CCA AGG ACC GAT CCA CAC T-3′); human TMPRSS4 reverse: (5′- GTG AAG TTG TCG AAA CAG GCA-3′); human CD147 forward: (5′-GTC TTC CTC CCC GAG CCC-3′); human CD147 reverse: (5′-GGT GGC ACG GAC TCT GAC-3′); human AXL forward: (5′-GTGGGCAACCCAGGGAATATC-3′); human AXL reverse: (5′-GTACTG TCCCGTGTCG GAAAG-3′).

### 2.6. Generation of CD147 Knockdown (KD) A549 Cell Line

The lentiviral CRISPR/Cas9 vector (pLentiCRISPR v2) and pLentiCRISPR with guide RNA (gRNA) targeting CD147 were purchased from and constructed by GenScript (Piscataway, NJ, USA). The sequence of gRNA that knocked down endogenous CD147 was CGTTGCACCGGTACTGGCCG. Lentiviral vectors with or without gRNA were transfected into HEK293T cells together with a packaging vector (psPAX2) and a VSV-G envelope-expressing plasmid was a gift from Didier Trono. Viruses were collected 72 h after transfection and used to infect A549 cells. A549 cells containing lentiviral vectors were selected with puromycin (8 μg/mL). Cell surface expression of CD147 in cells with the control vector or the vector with gRNA for CD147 was monitored by flow cytometry.

### 2.7. Western Blot Analysis

HEK293T cells transiently transfected with plasmids encoding spike proteins for 72 h were lysed on ice for 10 min in cell lysis buffer (Cell Signaling, Danvers, MA, USA) containing 1x Halt Protease inhibitor (ThermoFisher Scientific, Waltham, MA, USA), 1 mM PMSF, and 10 mM sodium fluoride. Proteins were analyzed using NuPAGE 8% Bis-Tris Protein Gels (Life Technologies, Carlsbad, CA, USA) and probed overnight at 4 °C with anti-SARS-CoV S protein antibody (NIH Biodefense and Emerging Infections Research Resources Repository, NIAID, NIH: Rabbit Sera Control Panels, Polyclonal Anti-SARS-CoV Spike Protein, NR-4569) at a 1:1000 dilution. Peroxidase-labeled, anti-rabbit secondary antibody (KPL, Gaitherburg, MD, USA) at a 1:5000 dilution was used for 1 h at room temperature followed by detection with a ProtoGlow ECL Kit (National Diagnostics). For detection of hACE2 proteins in different cell lines, whole cell extracts were prepared by solubilizing with a lysis buffer containing 20 mM Tris-HCl, pH 7.4, 150 mM NaCl, 1 mM EDTA, 1 mM EGTA, 1% Triton X-100, 2.5 mM sodium pyrophosphate, 1 mM β-glycerophosphate, 1 mM Na_3_VO_4_, 1 mM PMSF, 1 µg/mL leupeptin, and 1 µg/mL aprotinin. Protein concentrations were determined by BCA assay kit (ThermoFisher Scientific, Waltham, MA, USA). An equal amount of 3× SDS sample buffer containing 150 mM Tris-HCl, pH 6.8, 30% glycerol, 3% SDS, 1.5 mg/mL bromophenol blue dye, and 100 mM dithiothreitol was then added to each sample. Equal amounts of whole-cell extracts (50 μg of protein) and positive controls (Hela-hACE2, 293T-hACE2, 10 μg of protein) were separated on NuPAGE 4–20% Bis-Tris gels and transferred to nitrocellulose membranes. The membranes were blocked with 5% milk in 1×TBST for 1 h, probed with goat anti-human-ACE2 (AF933, Novus biologicals) at 1:1000 dilution at 4 °C for overnight, and incubated with a horseradish-linked secondary antibody at room temperature for 1 h. The protein bands were visualized with a chemiluminescence assay system (ThermoFisher Scientific, Waltham, MA, USA). The membranes were stripped and re-probed for β-actin, a constitutively expressed protein used as a loading control.

### 2.8. Flow Cytometry

To determine cell surface expression of human ACE2 receptor, cell lines were detached using cell stripper (Mediatech, Inc., Manassas, VA, USA) and collected according to the manufacturer’s suggestions. Cells were blocked in wash buffer (PBS, 2% FBS) on ice for 20 min and then stained with Zombie UV™ Dye (BioLegend, San Diego, CA, USA) for 20 min at room temperature, washed with wash buffer, and stained with human ACE-2 Alexa Fluor^®^ 488-conjugated Ab (clone 535919, FAB9332G, R&D system, Minneapolis, MN, USA ) or with an isotype control Ab, IgG2a-AF488, (0.5 μg per million cells) in 100 μL wash buffer on ice for 20 min. Cells were washed with wash buffer, fixed with 2% paraformaldehyde in PBS, and analyzed using a BD LSRFortessa™ X-20 Flow Cytometer (BD Biosciences, San Jose, CA, USA ). Results were analyzed with FlowJo (Tree Star Inc., Ashland, OR, USA).

### 2.9. Statistical Analysis

Statistical comparisons were performed using two-tailed Independent-Samples *t*-test using Prism 8 (GraphPad Software, LLC, San Diego, CA, USA); *p* < 0.05 was considered significant.

## 3. Results

### 3.1. Two-Plasmid HIV-1-Based Pseudotyped Viruses Expressing SARS-CoV-2 Spike Proteins

**Figure 1:**
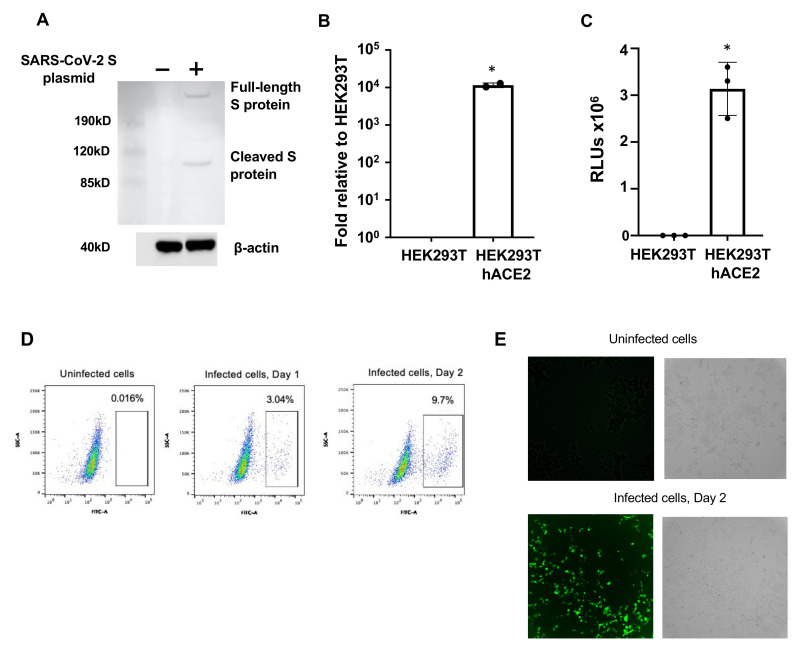
HIV pseudotyped virus expressing SARS-CoV-2 spike proteins. (**A**) HEK293T transfected with or without a SARS-CoV-2 spike-protein-expressing plasmid and the HIV vector pNL-Luc-R-E- for 48 h. Expression of SARS-CoV-2 spike proteins in cell lysates was determined by Western blot analysis as described in Materials and Methods. β-actin was used as a loading control. (**B**) Expression of hACE2 by HEK293T cells or hACE2-overexpressing HEK293T cells determined by RT-qPCR. Data are mean ± SD. * *p* < 0.05 between HEK293T with or without hACE2. (**C**) HEK293T and HEK293T-hACE2 cells were infected with HIV-pseudotyped SARS-CoV-2 viruses (~20 ng p24 per well; 48-well plate) for 1 h. Cells were washed and then cultured for 3 days before measurement of luciferase activity (RLUs) in infected cells. Data are mean ± SD. * *p* < 0.05 between HEK293T with or without hACE2. (**D**, **E**) HEK293T-hACE2 cells were infected with pseudotyped SARS-CoV-2 viruses with HIV vector containing GFP reporter gene. Infection was monitored by determining the GFP signal using flow cytometry (**D**) or a Bioteck Cystatin 5-cell imaging reader. (**E**) GFP and bright field images at 10× were captured.

We generated HIV pseudotyped luciferase virus particles expressing SARS-CoV-2 surface (spike, S) proteins based on the sequence of the Wuhan isolate [16]. The various systems of pseudotyped viruses used in this study are summarized in Table 1. We first generated pseudotyped viruses by transfecting an HIV vector without HIV accessary gene *vpr* (pNL-4-3 Luc R-E-), which is widely available from the NIH AIDS reagent program, along with a plasmid encoding full-length codon-optimized SARS-CoV-2 S into HEK293T cells in the presence of 10% FBS (Supplementary Figure S1). Viruses were collected 48 h after transfection. Expression of spike proteins in transfected cells was validated by anti-SARS S antibodies (Figure 1A). We infected HEK293T cells in the presence or absence of overexpressed hACE2 (Figure 1B) and found that infection of cells by pseudotyped virus expressing SARS-CoV-2 S proteins was hACE2-dependent (Figure 1C). Unlike published pseudotyped virus assays [11,18], DEAE dextran or polybrene was not needed. The pseudotyped SARS-CoV-2 viruses contained HIV p24 capsid protein, which can be used as a marker when studying viral attachment. The infectivity of pseudotyped viruses was monitored in the HeLa-hACE2-overexpressing cell line. When the HIV-1 vector expressing GFP was transfected together with the SARS-CoV-2 S plasmid (Table 1), infection by the pseudotyped virus was readily detectable by flow cytometry (Figure 1D) and by fluorescent microscopy (Figure 1E).

**Figure 2:**
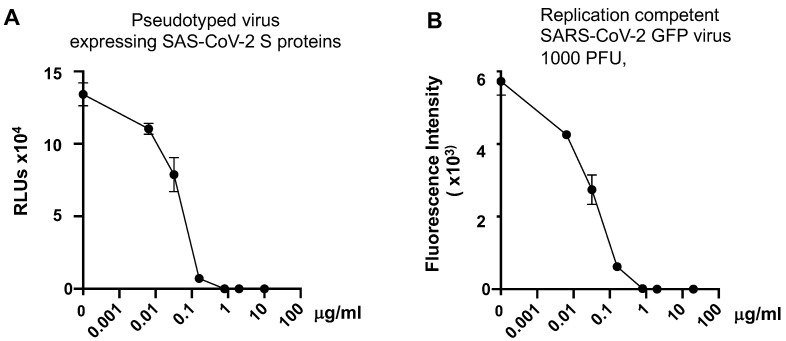
Neutralization of Pseudotyped SARS-CoV-2 viruses and replication-competent SARS-CoV-2 viruses by anti-spike RBD antibody. Pseudotyped SARS-CoV-2 viruses (**A**) and replication competent SARS-CoV-2 viruses expressing GFP (**B**) were incubated with different concentrations of monoclonal antibody against SARS-CoV-2 spike RBD proteins for 1 h before infection of HeLa-hACE2 cells and of Vero E6 cells, respectively. Luciferase activity or GFP signal in infected cells was measured at 48 h post-infection. Data are mean ± SD.

To determine whether pseudotyped viruses expressing SARS-CoV-2 S proteins were comparable to replication-competent SARS-CoV-2 viruses in neutralization assays, the anti-spike monoclonal antibody (mAb, Specifica clone #41) targeting the RBD domain at different concentrations was incubated with viruses for 1 h at 37 °C prior to infection of HeLa-hACE2 cells (for pseudotyped luciferase viruses) or Vero E6 cells (for replication-competent SARS-CoV-2 with mNeonGreen). After culturing cells for 48 h, infection was monitored by measuring luciferase activities or fluorescence as described in Materials and Methods. Pseudotyped SARS-CoV-2 and replication-competent viruses exhibited comparable sensitivity to the anti-spike RBD Ab in the neutralization assay. The 50 and 90 percent inhibitory doses (ID_50_, ID_90_) for pseudotyped viruses were 0.02 and 0.74 μg/mL, respectively, whereas the ID_50_ and ID_90_ for replication competent viruses were 0.02 and 0.89 μg/mL, respectively (Figure 2A, B).

### 3.2. Pseudotyped SARS-CoV-2 Viruses with HIV R- Vector under Serum-Free Conditions Infects a Broad Spectrum of Cell Types

**Figure 3:**
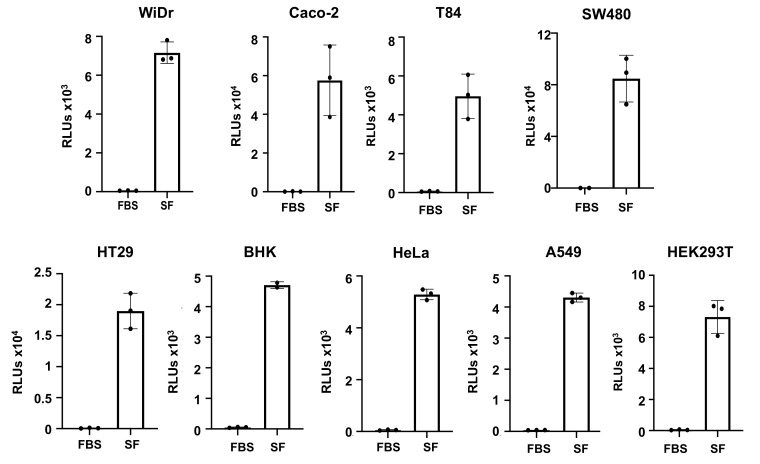
Serum-free, pseudotyped SARS-CoV-2 viruses (HIV R- vector) infect a broad spectrum of cell types. Pseudotyped SARS-CoV-2 viruses with the HIV R-vector were produced in the presence of 10% FBS (FBS on the left) or in the absence of serum (SF on the right). Viruses were collected 48 h after transfection and tested for ability to infect different cell lines. Infection was determined by measuring the luciferase activity at 48–72 h after infection. Data are mean ± SD.

Pseudotyped SARS-CoV-2 viruses with HIV-1 R-E- vector produced in media with 10% FBS infected hACE2-overexpressing cells (HEK293T-hACE2) but not cell lines without hACE2 overexpression (HEK293T in Figure 1C and various cell lines in Figure 3). We have previously generated serum-free, pseudotyped HIV viruses to study host factors sensitive to FBS [21]. HIV vector (pNL-4-3 Luc R-E-) and a plasmid carrying SARS-CoV-2 S proteins were transfected into HEK239T cells, and the medium was replaced with DMEM without serum 24 h after transfection. Viruses were collected 48 h after transfection. Pseudotyped viruses with HIV-1 R- vector prepared under serum-free conditions were able to infect HEK293T-hACE2 cells at high efficiency, and were able to infect various other cell lines, including intestinal cells (WiDr, Caco-2, T84, SW480, HT29), lung epithelial cells (A549), cervical epithelial cells (HeLa), and hamster or human kidney cells (BHK, HEK293T), albeit at a much lower efficiency (Figure 3). Infection did not occur when cells were infected with viruses without envelopes (Env-) or with viruses pseudotyped with HIV envelope (JR-FL), which requires CCR5 for viral entry (Supplementary Table S1), indicating the specificity of SARS-CoV-2 spike-protein-mediated entry of pseudotyped SARS-CoV-2 viruses.

**Figure 4:**
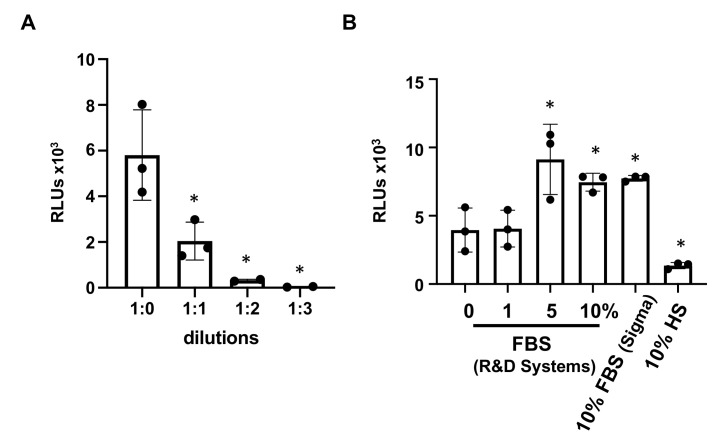
Infection of Caco-2 cells by pseudotyped SARS-CoV-2 virus (HIV R- vector) generated under serum-free conditions was not affected by the presence of FBS during viral attachment. (**A**) Caco-2 cells were infected by different dilutions of serum-free, pseudotyped SARS-CoV-2 viruses with the HIV R- vector. Differences between undiluted and diluted viruses were compared. (**B**) To determine whether FBS blocked the ability of pseudotyped SARS-CoV-2 viruses to infect Caco-2 cells, different concentrations of FBS from R&D Systems or 10% Sigma FBS or 10% human serum (HS) were present in serum-free viruses during viral attachment (1.5 h incubation). Infected cells were then cultured in medium containing 10% FBS. Infection was determined by measuring luciferase activity 72 h after infection. Differences between viruses treated with or without serum during viral attachment were compared. * *p* ± 0.05. Data are mean ± SD.

The infection of intestinal epithelial cells (Caco-2 cells) with endogenous hACE2 by serum-free pseudotyped SARS-CoV-2 viruses was dependent on the virus titer as indicated by the decreasing signal with serially diluted viruses (Figure 4A). To determine whether the presence of serum caused the inability of pseudotyped viruses prepared in 10% FBS to infect non-hACE overexpressing cells, FBS was added to serum-free virus stock to concentrations of 1, 5, and 10% before addition to intestinal epithelial cells. After 1.5 h attachment, infected cells were incubated with media with 10% FBS for three days before measuring luciferase activities. FBS from a different vendor as well as human serum (HS) were tested for comparison. We found that FBS promoted rather than inhibited infectivity of pseudotyped virus for Caco-2 cells, whereas HS inhibited infectivity of pseudotyped SARS-CoV-2 viruses (Figure 4B).

### 3.3. Pseudotyped SARS-CoV-2 Viruses with HIV R+ Vector Infect a Broad Spectrum of Cell Types

**Figure 5:**
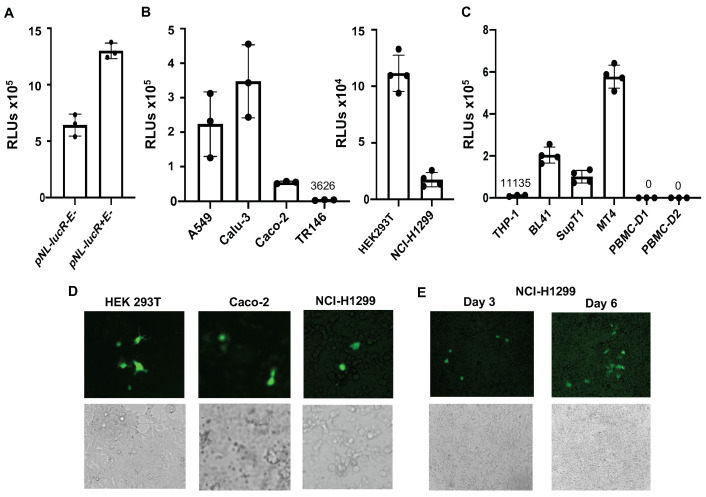
Pseudotyped SARS-CoV-2 viruses (HIV R+ vector) infect a broad spectrum of cell types. (**A**) Pseudotyped SARS-CoV-2 viruses were produced using pNL-luc R-E- (no Vpr) or pNL-luc R+E- (with Vpr) in the presence of 10% FBS during transfection. Viruses were used to infect HeLa-hACE2 cells. (**B**, **C**). HIV R+ pseudotyped SARS-CoV-2 viruses were used to infect adherent cells (**B**) or suspension cell lines (**C**). Activated PBMCs (from two donors, D1 and D2) were also included for comparison. Luciferase activity was determined 48–72 h after infection. Data are mean ± SD. Mean RLU values for TR146, THP-1, and PBMC infections are shown. No signal was detected in PBMCs after subtracting uninfected cell background (background RLU range: 50–70). (**D**) GFP+ pseudotyped SARS-CoV-2 viruses were used to infect HEK293T, Caco-2, and NCI-H1299 cells. The images at 10× were taken on day 4 post infection. (**E**) NCI-H1299 cells were infected with GFP+ pseudotyped SARS-CoV-2 viruses. Images at 10× were taken on day 3 and 6 post-infection.

Inclusion of the HIV *vpr* gene in the vector improves the efficiency of infection in specific cell types [22]; therefore, we generated pseudotyped SARS-CoV-2 viruses using an HIV vector with *vpr* (HIV R+). Viruses generated with the HIV R+ vector had higher luciferase readouts (Figure 5A) than those with the HIV R- vector. HIV R+ pseudotyped SARS-CoV-2 viruses produced in the presence of 10% FBS infected a broad spectrum of cell types, including HEK293T cells and lung (A549, Calu-3, NCI-H1299), intestinal (Caco-2), and oral (TR146) epithelial cells (Figure 5B). Additionally, the virus infected suspension cell lines, including BL41 (B cells), SupT1 and MT4 (T cells), and monocytic THP-1 cells (Figure 5C). We did not detect any signal in PHA-activated PBMCs (Figure 5C). Note that serum-free-generated HIV R+ pseudotyped SARS-CoV-2 viruses also infected multiple cell types (data not shown). A GFP-positive signal was detected in HEK293T, Caco-2, and NCI-H1299 cells infected with HIV R+ pseudotyped SARS-CoV-2 virus expressing GFP that was produced in the presence of 10% FBS (Figure 5D), although the frequency of GFP+ cells was low. Interestingly, we detected more GFP+ NCI-H1299 cells at day six post-infection than at day three post-infection (Figure 5E). Because cells were infected with pseudotyped viruses in a single-cycle infection assay, these results suggest that cell differentiation during culture supported viral gene expression at late time points after initial viral entry.

### 3.4. The Abundance of hACE2, TMPRSS2, and TMRPSS4 mRNAs Does not Correlate with the Viral Entry Profile in Cells without hACE2 Overexpression

**Figure 6:**
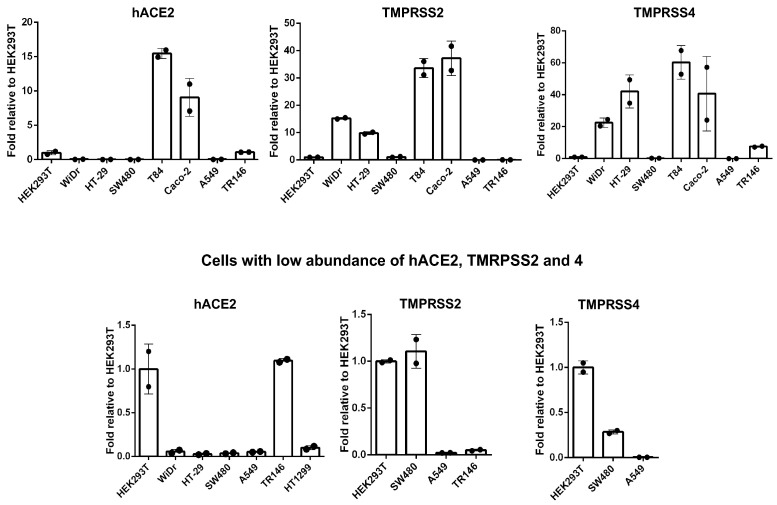
Gene expression of hACE2, TMPRSS2, and TMPRSS4 in colon, lung, and oral epithelial cell lines. Total RNA was extracted from colon (WiDr, HT-29, SW480, T84, and Caco-2), lung (A549 and NCI-HT1299), and oral (TR146) epithelial cells. Total RNA from HEK293T cells was included as a normalization control. Expression of hACE2, TMPRSS2, TMPRSS4, and GAPDH was determined by RT-qPCR and normalized using the corresponding data from HEK293T cells.

**Figure 7:**
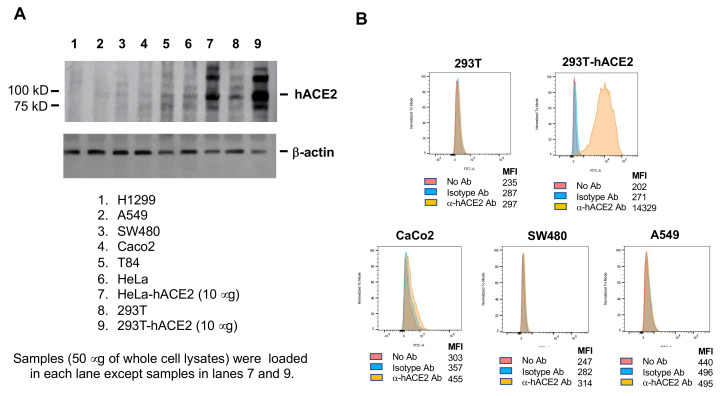
Protein and cell surface expression of hACE2 on cell lines with low and moderate hACE2 mRNA. (**A**) Whole cell lysates from cells with low vs. moderate hACE2 mRNA were fractionated on 4–20% NuPAGE, and hACE2 proteins were detected by Western blot analysis. The blot was stripped and reprobed with β-actin. HeLa, HeLa-hACE2, 293T, and 293T-hACE2 cells were included for comparison. (**B**) Cell surface expression of hACE2 was analyzed by flow cytometry. The specificity of anti-hACE2 Ab was assessed in HEK293T cells with or without hACE2 overexpression. Cell surface expression of hACE2 proteins on Caco-2, SW480, and A549 cells were determined. MFI, mean fluorescence intensity.

It has been suggested that hACE2 expression is a determinant of SARS-CoV-2 cell and tissue tropism. Although infection of ACE2-overexpressing cells by pseudotyped viruses clearly demonstrated hACE2-dependent viral entry, hACE2 mRNA levels were not associated with cell permissiveness in non-overexpressing cells (Figure 6). For example, the levels of hACE2 mRNAs in WiDr, HT29, and SW480 cells were much lower than in T84 and Caco-2 cells (Figure 6), but all of these intestinal cells were susceptible to pseudotyped SARS-CoV-2 viruses (Figure 3). Similarly, hACE2 mRNA was more abundant in TR146 cells than A549 cells (Figure 6), but the infection was more robust in A549 cells (Figure 5B), suggesting that the threshold for permissiveness is reached at fairly low hACE2 mRNA levels. Note that the lack of correlation of hACE2 mRNA with infection of different cell types was not due to the abundance of TMPRSS2 and TMPRSS4, which prime SASR-CoV-2 spike proteins for hACE2-dependent viral entry. For example, TMPRSS2 and TMPRSS4 mRNAs were more abundant in Caco-2 cells than A549 cells (Figure 6), but the infection was more pronounced in A549 (Figure 5B). We also examined hACE2 protein levels and cell surface expression among selected susceptible cell lines with very low or moderate hACE2 mRNAs (Figure 7). The results showed that HT1299 and A549 had no or low detectable levels of hACE2 proteins, whereas SW480, Caco-2, and T84 cells had detectable hACE2 (Figure 7A). Cell surface protein expression of hACE2, which would be more relevant for viral entry, was analyzed by flow cytometry. The specificity of anti-hACE2 Ab was shown in HEK293T cells with or without hACE2 overexpression (Figure 7B). Caco-2 cells expressed detectable levels of cell surface hACE2, whereas SW480 and A549 cells expressed nearly undetectable levels of cell surface hACE2 proteins (Figure 7B). Thus, similarly to results shown in Figure 6, with the exception of hACE2 overexpressing cells (HeLa-hACE2 or HEK293T-hACE2), levels of total protein or cell surface expression of hACE2 were not associated with infection profiles. For example, hACE2 proteins were detected in Caco-2 cells but not in A459 cells, but both cells were permissive for infection with pseudotyped SARS-CoV-2 viruses.

### 3.5. Differential Anti-Spike RBD mAb Sensitivity Indicates Different Entry Mechanisms

**Figure 8:**
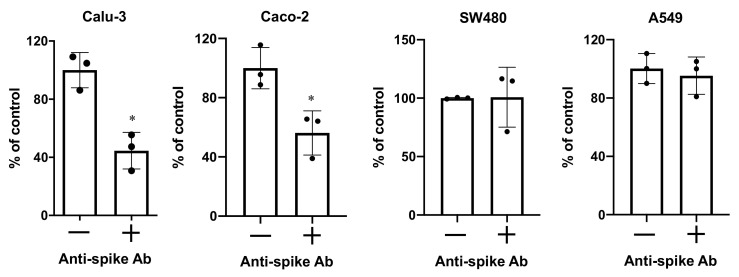
Anti-spike RBD mAb blocks pseudotyped SARS-CoV-2 viral entry in Calu-3 and Caco-2 but not in SW480 or A549 cells. Pseudotyped SARS-CoV-2 viruses (HIV R+ vector), produced in the presence of 10%FBS, were pre-incubated with or without anti-spike RBD mAb for 1 h at 37 °C before infecting target cells. Luciferase activity was determined 72 h after infection. Data are mean ± SD. Differences between anti-spike RBD mAb-treated viruses and untreated control were compared; * *p* < 0.05.

SARS-CoV-2 enters cells through binding to the hACE2 receptor via the spike RBD domain [23]. We determined whether anti-spike RBD mAb blocked infection of non-ACE-overexpressing cells. Pseudotyped SARS-CoV-2 viruses were pre-incubated with anti-spike RBD mAb at 10 μg/mL at 37 °C for 1 h before infection of cells. Viral infection was determined 48–72 h after infection. We found that anti-spike RBD mAb treatment resulted in approximately 50% inhibition of infection of lung epithelial Calu-3 cells and intestinal epithelial Caco-2 cells, which are known to express a high abundance of hACE2 [1], whereas anti-spike RBD mAb did not exhibit any anti-viral activity in intestinal epithelial SW480 cells or lung epithelial A549 cells (Figure 8), which had a low abundance of hACE2 compared to Caco-2 cells (Figure 6 and Figure 7B). Note that much higher concentrations of anti-spike RBD mAb were required to suppress viral infection in Calu-3 or Caco-2 cells compared to HeLa-hACE2 cells (Figure 2A).

**Figure 9:**
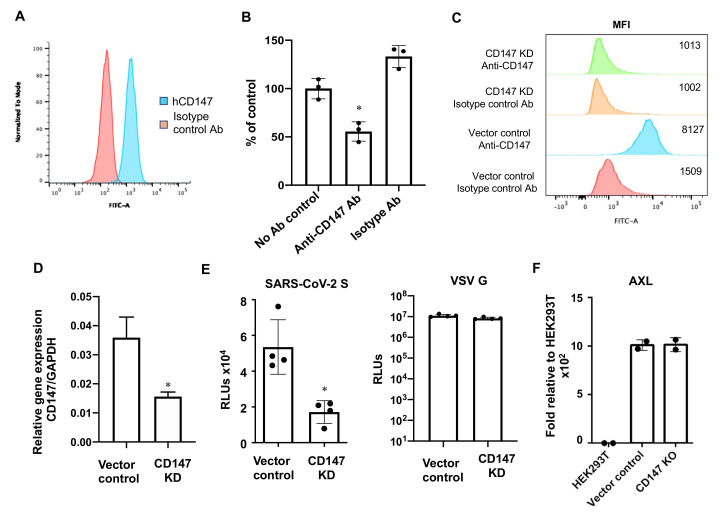
CD147 is involved in SARS-CoV-2 entry in A549 cells. (**A**) Cell surface expression of CD147 in A549 cells was determined by flow cytometry. (**B**) A549 cells were incubated with 10 μg/mL of anti-CD147 mAb or isotype control Ab for 1 h before exposure to pseudotyped SARS-CoV-2 viruses. Luciferase activity was determined 72 h after infection. (**C**) Cell surface expression of CD147 was determined in A549 cells with CD147KD or with vector alone to confirm the absence of CD147 in the CD147KD cells. (**D**) CD147 gene expression in CD147-expressing control cells and CD147KD cells was determined by RT-qPCR. (**E**) A549 cells with or without CD147KD were infected with pseudotyped viruses with SARS-CoV-2 spike or VSVG proteins. Infection was determined by measuring the luciferase activity 72 h after infection. (**F**) Expression of AXL in A549 cells with CD147KD or vector alone was determined by RT-PCR. HEK293T cells were included as a comparison. Data are mean ± SD; * *p* < 0.05.

CD147, known as *basigin* or EMMPRIN, has been suggested as an alternative receptor for SARS-CoV-2 entry [24]. We examined the involvement of CD147 in hACE2-independent viral entry of A549 cells. We confirmed gene expression of CD147 in A549 cells by RT-qPCR (Supplementary Figure S2) and cell surface expression by flow cytometry (Figure 9A). We found that anti-CD147 mAb reduced infection of A549 cells by pseudotyped SARS-CoV-2 viruses (Figure 9B). We then generated CD147 knockdown (KD) A549 cells using CRISPR/Cas9 technology. Down-regulation of CD147 in A549 CD147KD cells was confirmed by flow cytometry (Figure 9C) and by RT-qPCR (Figure 9D). We then infected stable CD147KD cells with pseudotyped SARS-CoV-2 viruses and found that infection of CD147KD cells decreased by 68% compared to infection of cells with the vector control (Figure 9E, left panel), indicating the involvement of CD147 in SARS-CoV-2 viral entry. Note that infection of cells with the vector control and CD147KD cells by viruses pseudotyped with VSV G proteins was comparable (Figure 9E, right panel), indicating the specificity of SARS-CoV-2 spike-protein-mediated entry.

Neither neutralizing antibody against CD147 nor knocking down CD147 completely abolished infection of A549 cells by pseudotyped SARS-CoV-2 viruses (Figure 9B,E). The tyrosine-protein kinase receptor AXL has been shown to be an alternative receptor that mediates SARS-CoV-2 infection of lung epithelial cells through interactions with the N-terminal domain of SARS-CoV-2 spike proteins [25]. We determined the expression of AXL in A549 cells with CD147 KD or mock KD and found that high levels of AXL were expressed in both lines (Figure 9F), suggesting that AXL may contribute to the infection of CD147 KD cells. We then determined gene expression profiles of CD147 and AXL in various cell lines and found that CD147 was detectable in all cell lines with the lowest level in TR146 oral epithelial cells (Supplementary Figure S2). AXL was expressed with the highest abundance by NCI-H1299 cells followed by TR146, SW480, and A549 cells (Supplementary Figure S2). NCI-H1299, SW480, and A549 cells were more susceptible to infection by pseudotyped SARS-CoV-2 virus than TR146 cells, indicating that the receptor usage for SARS-CoV-2 entry is complex. Nevertheless, this pseudotyped SARS-CoV-2 virus system will be useful for exploring new receptors in addition to anti-viral screening in cells without hACE2 overexpression.

## 4. Discussion

Several systems of pseudotyped viruses expressing SARS-CoV-2 S proteins have been developed. Cells that overexpress hACE2 receptors, or cell lines with naturally high abundance of hACE2 receptors, have provided insight into viral entry and are useful for screening neutralizing Abs [1,9,10,11,12,13,14]. In this report, we show that HIV vector-based, pseudotyped viruses expressing SARS-CoV-2 S proteins could infect a broad spectrum of cell lines. The neutralizing activities of mAb against pseudotyped SARS-CoV-2 viruses and against replication-competent viruses were comparable. The infection assay did not require polybrene or modified spike proteins to enhance viral infection. Pseudotyped SARS-CoV-2 viruses with an HIV R- vector that were produced under serum-free conditions or pseudotyped viruses with HIV R+ vector, regardless of the presence or absence of FBS, infected intestinal, lung, cervical epithelial cells, and T- and B-cell lines but not activated PBMCs. Although the ability of pseudotyped SARS-CoV-2 virus to enter cells is not equivalent to productive virus replication in these cell lines, cellular functions may be altered upon viral entry. We found that anti-spike RBD mAb blocked viral entry in Calu-3 or Caco-2 cells with high levels of hACE2 but did not block entry in SW480 or A549 cells with low abundance of hACE2. We further showed that CD147 was involved in viral entry in A549 cells.

We discovered that pseudotyped SARS-CoV-2 viruses with HIV R- vector produced under serum-free conditions or viruses with an HIV R+ vector possessed increased ability to enter many cell types. Although the mechanisms by which serum-free culture conditions changed the virus infection profile remain to be determined, the addition of FBS during the infection did not block the infection of Caco-2 cells by serum-free viruses. In fact, addition of FBS to serum-free viruses during the attachment slightly promoted viral infection (Figure 4B). FBS contains various glycosaminoglycans [26], which may affect viral infection. Increases in virus infectivity in serum-free cultures or in the presence of different types of sera have been reported for human hepatitis C viruses and foot-and-mouth disease viruses; properties of the virus have been found to differ depending on the culture medium used during virus preparation [27,28,29]. It remains to be determined whether viral production conditions affect the structure of spike proteins presented on the virions that, in turn, impact their infectivity.

Vpr, a multifunctional protein, can be incorporated into the virus particles, and is known to modulate cell cycle and transcription [30,31]. Vpr is also involved in the process of virus maturation (budding into cytoplasmic vacuoles or from the plasma membrane) [32]. Vpr improves infection of HIV in primary macrophages [22]. It remains to be determined how an HIV R+ vector broadens the spectrum of permissive cell types for infection with pseudotyped SARS-CoV-2 viruses and why the vector with vpr removes the requirement for serum-free conditions. It is possible that viruses with or without Vpr bud out from different membranes, as seen in HIV [32]. Vpr may also modulate host factors that are packaged in the virions. Fortunately, we have the means to explore the viral entry event in various cell lines using pseudotyped viruses, and we are not limited to studies in cells that overexpress hACE2.

**Figure 10:**
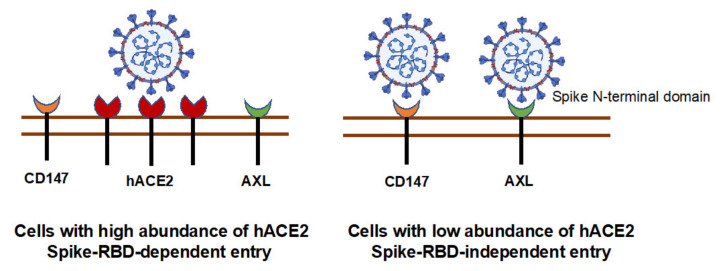
Involvement of hACE2, CD147, and AXL in SARS-CoV-2 entry. hACE2 is a dominant receptor for viral entry when target cells express high abundance hACE2. In cells with low abundance or no hACE2, an alternative receptor (e.g., CD147) is used for the viral entry. AXL has been shown to promote SARS-CoV-2 viral entry in lung epithelial cells via interacting with N-terminal domain of spike proteins [25].

SARS-CoV-2 RNA has been detected in various tissues [33,34]. hACE2 mRNA is expressed in a wide variety of tissues and cell types, although hACE2 mRNA expression in the lung is not high [6,35]. Several alternative receptors including CD147 and AXL have been proposed to mediate viral entry [25,36]. The data regarding the role of CD147 as a receptor for SARS-CoV-2 entry has been inconsistent [24,37]. Wang et al. showed that CD147 interacts with spike RBD proteins and mediates SARS-CoV-2 viral entry in vitro and in infection of mice expressing CD147 [24]. In contrast, a study by Shilts did not observe binding of full-length spike protein to CD147. Knocking down CD147 in Calu-3 cells, which express high levels of hACE2, did not impact the susceptibility of cells to SARS-CoV-2 viral infection. We found that a mAb targeting the spike RBD domain did not block viral entry in SW480 and A549 cells, which expressed CD147 but low abundance of hACE2 (Figure 8 and Supplementary Figure S2), suggesting that the RBD domain did not interact with CD147 and did not directly mediate infection in these cells. However, anti-CD147 mAb suppressed viral infection of A549 cells, indicating a role for CD147 in viral entry. Reduced infection of A549 CD147KD cells further implicates CD147 as a mediator of viral entry. On balance, therefore, it appears that CD147 acts as an alternative entry receptor in cells with no or low abundance of hACE2 but that the spike RBD domain-mediated entry is unlikely to involve interaction with CD147 (Figure 10). CD147 is involved in the process of macropinocytosis [38], which is an actin-mediated, clathrin-independent endocytosis important for viral entry [39]. CD147 has been shown to promote entry of pentamer-expressing human cytomegalovirus into epithelial and endothelial cells through macropinocytosis [40]. Thus, studies regarding its role in SARS-CoV-2 viral entry via macropinocytosis in cells with low abundance of hACE2 will likely provide insight into SARS-CoV-2 pathogenesis.

A549 expresses nearly undetectable levels of hACE2. Anti-CD147 Ab or knockdown of CD147 did not completely suppress SARS-CoV-2 infection (Figure 9), suggesting the virus may enter cells via AXL, which was expressed at significant levels in CD147 KD cells. Future studies involving knocking down AXL in A549 CD147 KD cells and co-expressing various alternative receptors in hACE2 null cells promise to provide a better understanding of whether these receptors act independently or in concert for infection.

In summary, we report viral entry of pseudotyped SARS-CoV-2 viruses into a broad spectrum of cell types via hACE2-dependent and -independent mechanisms. This simple, two-plasmid system offers a useful tool for understanding viral entry events of SARS-CoV-2 and for drug screening in non-hACE2-overexpressing cell lines. Whereas these studies confirmed the general model of a key role for the interaction of RBD and hACE-2 in cells expressing a high level of this receptor, the results suggest that the interaction of a different region of the spike protein with CD147 may be involved in the infection of epithelial and other cells expressing low levels of hACE2.

## Figures and Tables

**Table 1:** Summary of pseudotyped SARS-CoV-2 viruses in this study.

Plasmids (Spike and HIV Vector)	Culture Condition during Transfection	hACE2-Overexpressing Cell Lines	Cells without hACE2 Overexpression
pcDNA3.1-Spike, pNL-Luc R-E-	10% FBS	Yes	No
pcDNA3.1-Spike, pNL-Luc R-E-	Serum-free	Yes	Yes
pcDNA3.1-Spike, pNL-Luc R+E-	10%FBS	Yes	Yes
pcDNA3.1-Spike, pNL-Luc R+E-	Serum-free	Yes	Yes
pcDNA3.1-Spike, pLai3ΔenvGFP3	10%FBS	Yes	Yes

## Data Availability

Not applicable.

## Supplementary Materials

The following are available online at https://www.mdpi.com/article/10.3390/v13060953/s1, Figure S1. A diagram of generation of pseudotyped luciferase viruses expressing SARS-CoV-2 S proteins in the study, Figure S2. Gene expression of CD147 and AXL in different cell lines, Table S1. Luciferase activities of cells infected by pseudotyped viruses without envelopes or HIV-1 (JR-FL) envelope.
