# Supplementary material for: Human Immunodeficiency Viruses Pseudotyped with SARS-CoV-2 Spike Proteins Infect a Broad Spectrum of Human Cell Lines through Multiple Entry Mechanisms
Source: Viruses. 2021 May 21;13(6):953. doi: 10.3390/v13060953 (PMC8224355; doi:10.3390/v13060953)
Supplement: Supplementary file 1 [file viruses-13-00953-s001.zip › viruses-1166133-supplementary.pdf]

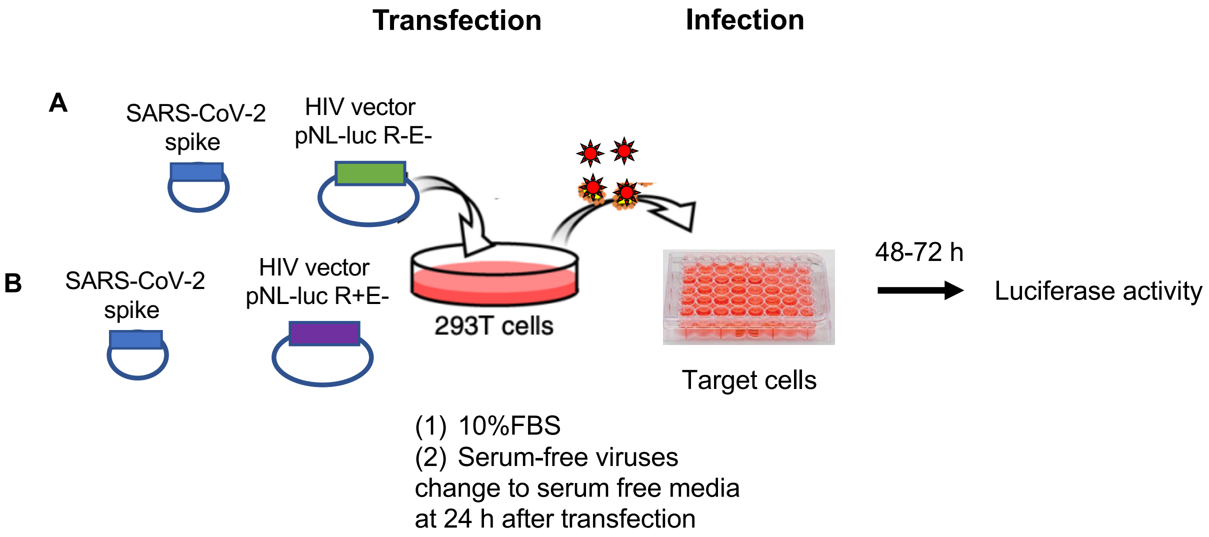

Figure S1. A diagram of generation of pseudotyped luciferase viruses expressing SARS-CoV-2 S proteins in the study.

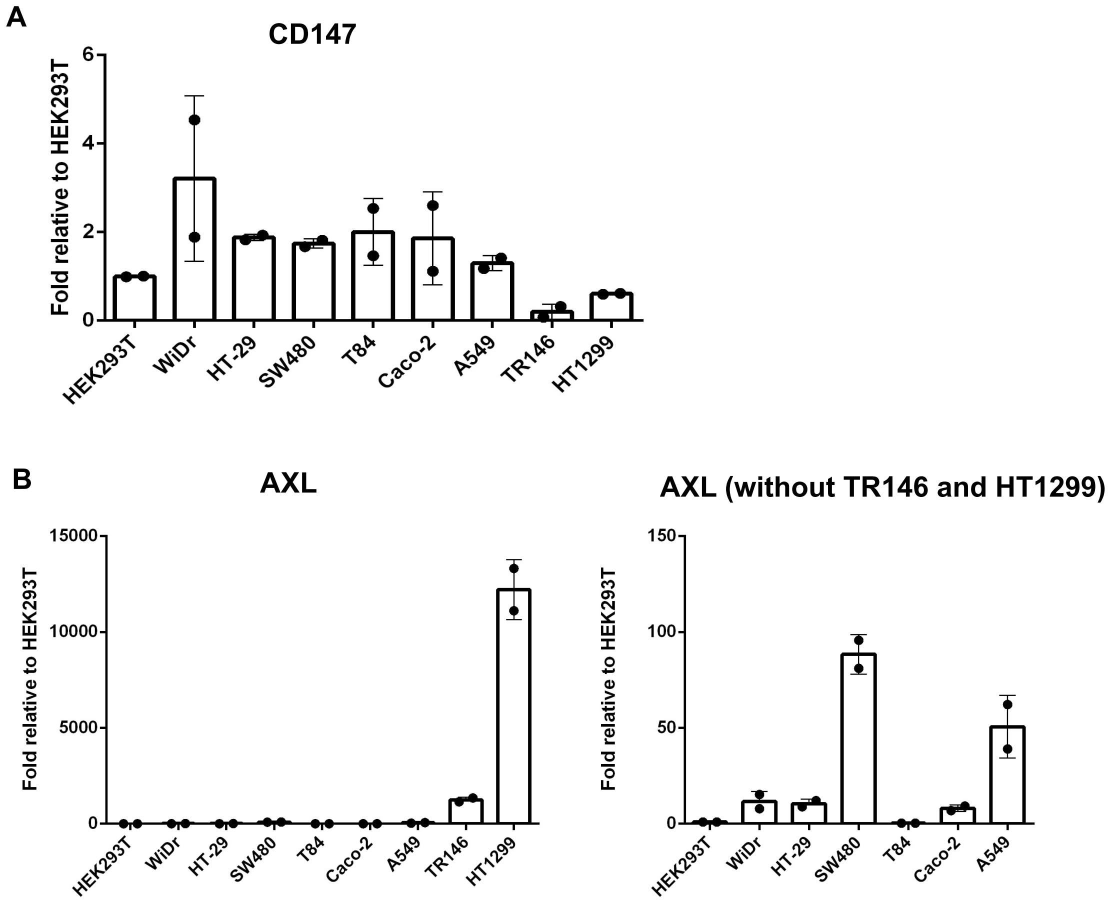

Figure S2. Gene expression of CD147 and AXL in different cell lines.

Cell types	No infection	Env (-) virus	HIV-1 (JR-FL)
Caco-2	27.6+/-5.0	26.7+/-6.5	34.3+/-5.1
293T	37.7+/-13.6	44.7+/-6.8	42.0+/-10.1
A549	23.3+/-9.0	50.0+/-3.0	38.0+/-11.5
NCI-HT1299	31.0+/-2.3	44.3 +/-18.0	42.3+/-8.1
HeLa	50.0+/-2.0	46.6 +/-16.0	48+/- 20.1

Table S1. Luciferase activities of cells infected by pseudotyped viruses without envelopes or HIV-1 (JR-FL) envelope. Samples from uninfected cells were included as the background.
